# Parliament and the Professions

**Published:** 1921-04-23

**Authors:** 


### Parliament and the Professions.

### Pensioners with Heart Disease.

The Minister of Pensions stated that a sum of nearly £4,000,000 a year is at present being paid to men who are pensioned for heart disease. Every effort is being made by the establishment of Cardialogical Clinics with improved methods of diagnosis and treatment to restore the health of men who suffer from this complaint.

### Ministry of Health Salaries.

Sir Alfred Mond, in answer to a question, stated that the total number of medical men on the staff of the Ministry of Health (including two part-time specialists) is 100, namely, sixty-three at headquarters and thirty-seven in regional offices. The salaries of the headquarters staff amount to £51,549, and the war bonus to £31,681, being a total cost of £83,230. The salaries of the regional officers amount to £47,000. The Local Government Board medical officers in 1914 were thirty-one, and the salaries in that year were £20,571.

### Encephalitis Lethargica.

The Minister of Health stated that 1,572 cases of encephalitis lethargica (sleeping-sickness) have been reported in England and Wales between April 1, 1920, and March 31, 1921. The deaths, so far as is known at present, amount to 495 in the same period. The disease was made compulsorily notifiable on January 1, 1919, and since then special reports have been received from medical officers of health on practically all cases, while special inquiries have been made and advice given by medical officers of the Ministry in suitable cases. The pathology of the disease is being studied in association with the Medical Research Council.

